# Transcriptome response analysis of *Arabidopsis thaliana* to leafminer (*Liriomyza huidobrensis*)

**DOI:** 10.1186/1471-2229-12-234

**Published:** 2012-12-11

**Authors:** Sufang Zhang, Zhen Zhang, Le Kang

**Affiliations:** 1 State Key Laboratory of Integrated Management of Pest Insects and Rodents, Institute of Zoology, Chinese Academy of Sciences, Beijing, China; 2 Key Laboratory of Forest Protection, Research Institute of Forest Ecology, Environment and Protection, Chinese Academy of Forestry, State Forestry Administration, Beijing, China

**Keywords:** Plant defenses, Transcriptome, Microarray, Leaf miner, Pathogen, Systemic defense

## Abstract

**Background:**

Plants have evolved a complicated resistance system and exhibit a variety of defense patterns in response to different attackers. Previous studies have shown that responses of plants to chewing insects and phloem-feeding insects are significantly different. Less is known, however, regarding molecular responses to leafminer insects. To investigate plant transcriptome response to leafminers, we selected the leafminer *Liriomyza huidobrensis*, which has a special feeding pattern more similar to pathogen damage than that of chewing insects, as a model insect, and *Arabidopsis thaliana* as a response plant.

**Results:**

We first investigated local and systemic responses of *A. thaliana* to leafminer feeding using an Affymetrix ATH1 genome array. Genes related to metabolic processes and stimulus responses were highly regulated. Most systemically-induced genes formed a subset of the local response genes. We then downloaded gene expression data from online databases and used hierarchical clustering to explore relationships among gene expression patterns in *A. thaliana* damaged by different attackers.

**Conclusions:**

Our results demonstrate that plant response patterns are strongly coupled to damage patterns of attackers.

## Background

Plants have evolved a complicated resistance system to defend against damages from various types of attackers. Based on many studies devoted to plant defense signal transduction, three main plant defense hormones have been identified. They are salicylic acid (SA), jasmonic acid (JA), and ethylene (ET), which are the key signal molecules involved in defense against pathogens, insects, and fungi, respectively [1-3]. It has recently been shown, however, that these signals are correlated in a very complex fashion; sometimes they conflict, while at other times they cooperate [4,5], indicating that plants express various defense patterns when damaged by different attackers. The factors determining these plant response patterns are still not clear, however.

Plant responses to chewing insects and phloem-feeding insects are significantly different [6,7]. The two types of insects not only produce different elicitors, but also have different feeding guides. For example, wounding leads to leakage of plant cellular liquids, stimulating the mobilization of many defense pathways [8]; insect feeding causes similar damage to plants, but the elicitors in insect saliva can induce special plant defense proteins [9] or conversely suppress plant defense signals [10]. Phloem-feeding insects cause little wounding but have long damage durations, and plant defenses to these insects are thus slight [11,12]. In comparison to chewing insects and phloem-feeding insects, little is known about molecular responses to leafminers, which are insects with special feeding guides.

Pea leafminers (*Liriomyza huidobrensis*) feed on over 100 species in 22 plant families, including the model plant *Arabidopsis thaliana*. During the adult stage, the female fly uses her ovipositor to penetrate the epidermis of host plant leaves; she then either lays eggs inside the leaves or feeds at the wound site, which can greatly reduce photosynthesis and eventually kill young plants [13]. Although male flies are unable to puncture leaves, they occasionally feed at the wounds and oviposition punctures made available by females [14]. Plant cells around the oviposition holes usually die and form ayellow necrotic spot, which is similar to necrotic spots created by pathogens [15]. The leafminer is thus a special insect with a damage pattern in plants somewhat similar to pathogens, and is a good model to test relationships between insect damage patterns and plant defense patterns. In addition to its importance as a model insect in plant defense studies, this pest can cause economic losses to host plant crops, as mining larvae consume foliage while dwelling inside leaves [16]. Leafminer larvae consume mesophyll both in palisade and spongy tissues [13,17]. Leafminer plant damage is very serious, but the insects are difficult to detect at early stages because they are well-hidden in the leaves; it is therefore important to explore the inherent defenses of plants to this insect. Consequently, our study of leafminer-plant interactions is not only important for exploring the mechanisms of plant-insect interactions, but also of value for leafminer pest management.

In this study, we used an Affymetrix ATH1 *A. thaliana* microarray, from an organism with a well-understood genomic background and thus capable of comprehensively representing transcriptome response, to study expression pattern changes in *A. thaliana* in response to both local (LI) and systemic (SI) pea leafminer damage. We found that more than 3000 genes were induced in the locally-damaged tissue, and that these genes could be divided into two categories: metabolic processes and stimulus response. Systemic defense of *A. thaliana* to pea leafminer was very similar to local defense, and the SI-induced genes were almost the same as LI-induced genes, but fewer in number and with lower fold changes. Our analysis of defense signal pathways to leafminer in *A. thaliana* revealed that signal responses to insects, bacteria, and fungi were all greatly induced. We then downloaded data from online databases and used hierarchical clustering to explore the relationships among *A. thaliana* expression patterns induced by different types of predators. Interestingly, two different types of data provided evidence that the response to pea leafminer in *A. thaliana* is more similar to that induced by pathogens than by insects, supporting our hypothesis that plant response patterns are closely related to the damage pattern of attackers.

## Results

### Microarray expression patterns in leafminer-damaged *A. thaliana*

**Figure 1:**
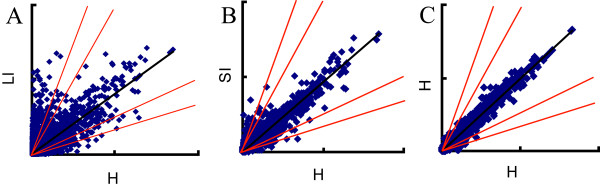
**Expression patterns of untreated and treated *Arabidopsis thaliana *plants.** Locally-damaged (LI) and systematically-damaged (SI) samples compared with corresponding untreated controls (H). A negative control hybridization was carried out using two untreated control samples. Diagonal red lines represent 2-fold and 3-fold induction/repression ratio cutoffs relative to the best fit line through the normalized data (middle black line).

**Figure 2:**
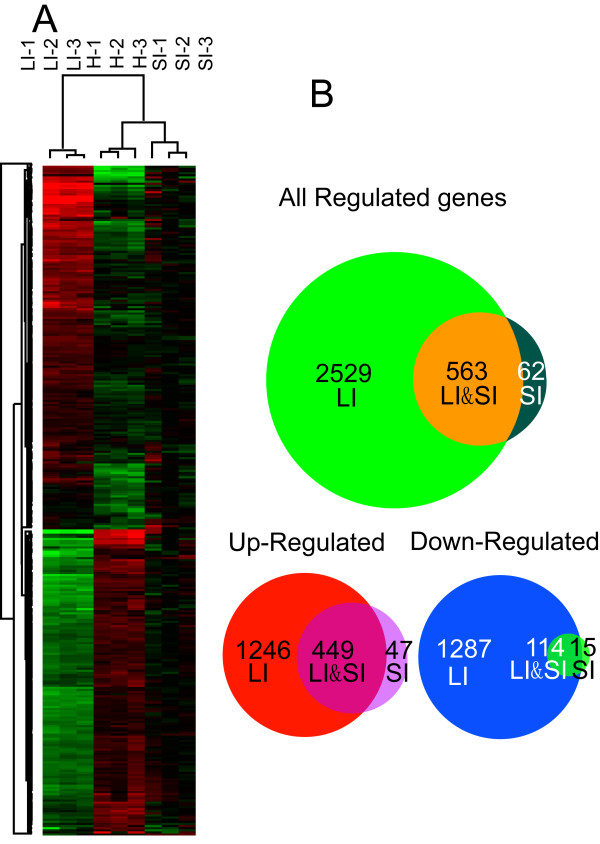
**Local and systemic response of *A. thaliana *to leafminer damage.** (**A**) Cluster analysis of local and systemic response of *A. thaliana* to leafminer damage. (**B**) Venn diagrams of local and systemic response of *A. thaliana* to leafminer damage.

To evaluate expression pattern changes caused by leafminer damage in *A. thaliana*, we used an Affymetrix ATH1 *A. thaliana* GeneChip, which contained 22,810 probe sets covering most identified cDNA and open reading frames. Three biological replicate experiments were performed with eight plants per treatment. Using RNA extracted from the three biological replicates, cDNAs were synthesized and hybridized to three replicate ATH1 GeneChips. To identify genes significantly regulated by instar feeding, the data were normalized and subjected to a significance analysis of microarrays (SAM). The quality and reproducibility of the data among the three experiments was examined by comparing all probe sets identified as “present.” The locally-infected (LI) tissues differed greatly from the control (healthy) ones, whereas the systemically-infected (SI) tissues accorded well with the controls (Figure 1). A comparison plot of two different control experiments (Figure 1C) indicated that our experiments were consistent among different samples. Another analysis also confirmed that different samples from plants subjected to the same treatment had great coherence, because duplicates of the same treatments clustered together (Figure 2A). LI and SI expression patterns differed greatly from one another (Figure 2A). Approximately 3096 genes were identified from LI tissue, with 1695 up-regulated and 1401 repressed; a much smaller number of genes were identified from the SI dataset, which contained 625 differentially-expressed genes, of which 496 were up-regulated and 129 were down-regulated. Quantitative real-time PCR (qRT-PCR) was used to validate the microarray hybridization data, with the results confirming the microarray epxeriment’s reliability (Additional file 1).

Further analysis indicated a high correlation between differentially-expressed genes from SI and LI samples. Although LI and SI expression patterns were very different (Figure 2A), 567 of the 625 genes regulated in SI were differentially-regulated in LI (Figure 2B). The up- and down-regulated genes of SI, with a few exceptions, comprised a subset of the genes differentially-regulated in LI (Figure 2C, D). These results indicate that systemic and local defense responses of *A. thaliana* to leafminers involve similar mechanisms, but with different gene expression fold change ranges. A GO analysis of genes exclusively regulated in SI revealed that the 47 genes exclusively up-regulated in SI fell into classes focused on transcriptional regulation and stimulus response to elicitors such as hormones and chitin (Additional file 2).

### Functional classification of genes regulated by leafminer in *A. thaliana*

**Figure 3:**
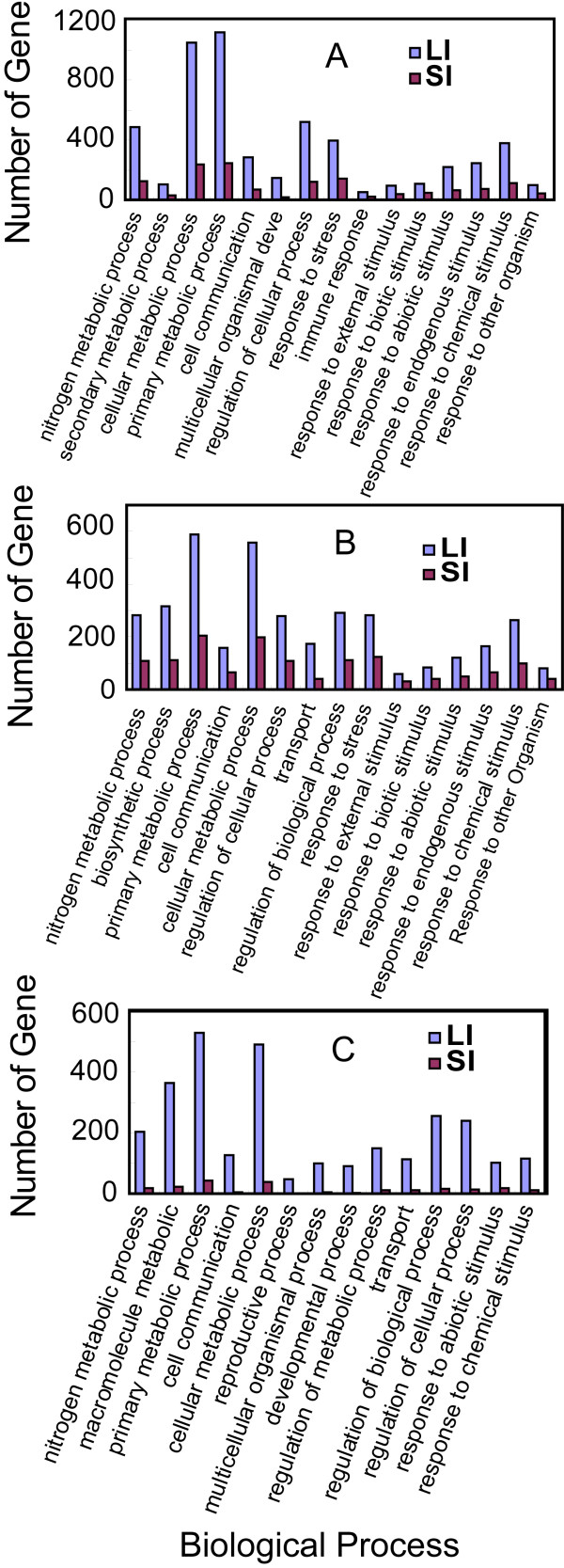
**GO classification of genes expressed in response to local and systemic leafminer damage in *A. thaliana *(*P *<0.01).** (**A**) All expressed genes. (**B**) Up-regulated genes. (**C**) Down-regulated genes. Only categories with more than 50 unigene clusters in LI are included.

Biological process analysis of differentially-expressed genes using WEGO [18] classified LI- and SI-regulated genes into similar categories, but with more genes numbers in each biological process category in LI samples (Figure 3). The entire set of LI- and SI-regulated genes was concentrated into two large categories: metabolism and stress response (Figure 3A). Nitrogen, secondary, cellular, and primary metabolic processes were all significantly represented. Another process worthy of mention was regulation of cellular processes, which accounted for nearly 1/6 of the entire set of regulated genes. Many stimulus response genes were regulated by leafminer damage, the most significant categories being response to endogenous stimulus and response to chemical stimulus. These categories represented the majority of stimulus response genes expressed in both LI and SI. Functional classifications for up-regulated genes were largely consistent with those of all regulated genes, although the differences in the numbers of genes in each GO category between LI and SI were smaller for up-regulated genes than for all regulated ones (Figure 3B). Down-regulated genes were mainly related to metabolism, with a few genes related to stimulus response (Figure 3C).

Differentially-expressed genes in both LI and SI were related to metabolism and stimulus response, but the question might be asked as to whether up-regulated genes regulate different subpathways than the down-regulated ones. To determine this, we dissected pathways regulated by genes up- and down-regulated by leafminer damage using EasyGO, a Gene Ontology-based annotation and functional enrichment analysis tool [19]. Nearly all the up-regulated genes locally and systemically expressed in leafminer-damaged *A. thaliana* were clearly related to defense (Additional files 3 and 4). Although we used a very strict cutoff (*P* = 1 × 10^-5^), we found that many biological processes were enriched in up-regulated genes of LI and SI. Pathways directly related to defense were dramatically up-regulated, including responses to other organisms such as bacteria and fungi, wounding, abiotic stimuli such as osmotic stress, water, and cold, and chemical stimuli, including JA, ET, and chitin. Even metabolic processes that were enriched were related to stimulus response, such as cellular aromatic compound metabolic processes, typically indole and derivative metabolic processes. Response to abscisic acid stimulus and amino acid derivative metabolic processes were markedly up-regulated specifically in LI (Additional file 3). Down-regulated pathways were not as dramatically regulated as the up-regulated pathways (Additional files 5 and 6). Using less stringent cutoffs (*P*=0.00001 for LI, *P*=0.001 for SI) than those used in analysis of up-regulated genes, we found that the pathways of LI down-regulated genes were concentrated in the categories of cell surface receptor linked signal transduction, pigment biosynthetic processes, response to auxin stimulus, and response to some abiotic stimuli, such as heat and light (Additional file 5); SI down-regulated genes were enriched in response to temperature stimulus, secondary metabolic processes, and amino acid and derivative metabolic processes, the latter of which was similar to the locally down-regulated pathways (Additional file 6).

### Comparison of expression patterns in *A. thaliana* damaged by leafminer and other typical plant damage organisms

Functional analysis of differentially-expressed genes in leafminer-damaged *A. thaliana* revealed, in addition to regulation of wounding and abiotic stimulus response genes, that many pathogen defense genes were also up-regulated, indicating that leafminers are different from other insects. Leafminers were already known to be unusual because their damage pattern is similar to that of pathogens. The question then arose: is the expression pattern from leafminer-damaged *A. thaliana* more similar to that of *A. thaliana* damaged by pathogens or damaged by other insects?

**Figure 4:**
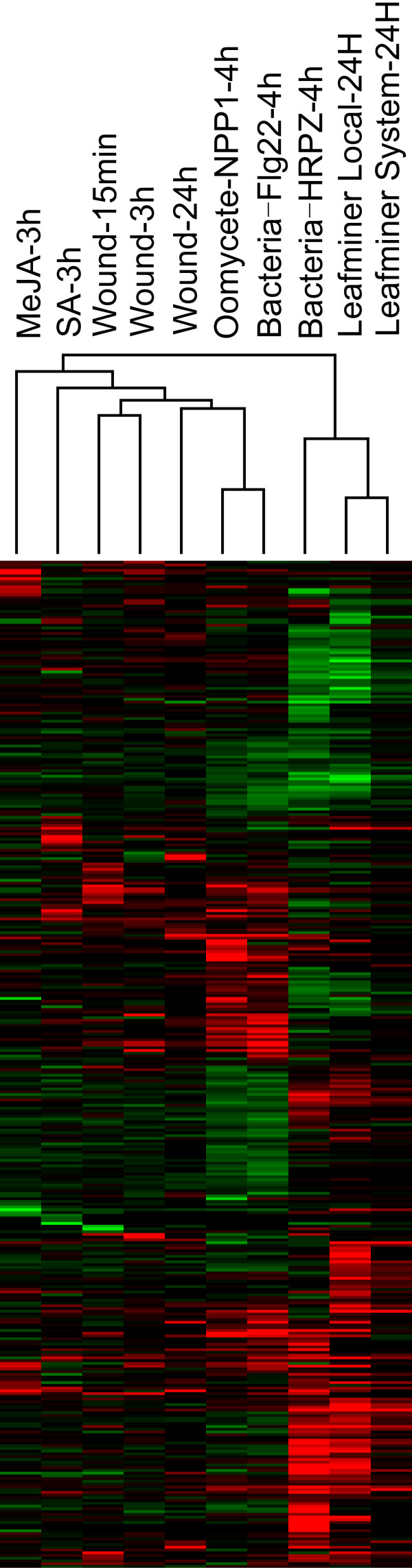
**Hierarchical clustering of *A. thaliana *differentially-expressed genes induced by leafminers and other elicitors.**

To answer this question, we performed hierarchical clustering on differentially-expressed genes in *A. thaliana* stimulated by leafminer and eight other factors involving wounding, pathogen specific elicitors, and plant hormones such as MeJA (methyl jasmonate) and SA. Data were downloaded from the TAIR database (ftp://ftp.arabidopsis.org/home/tair/Microarrays/Datasets/), and differentially-expressed genes were extracted using SAM as with our experiment data. Cluster analysis revealed that leafminer-induced expression patterns were most similar to those produced by the type-III section system (TTSS) bacterial elicitor HRPZ, and very different from patterns produced by stimuli such as wounding and MeJA. The observed clustering results support the hypothesis that leafminer-damaged *A. thaliana* expression patterns are strongly influenced by leafminer damage patterns (Figure 4).

**Figure 5:**
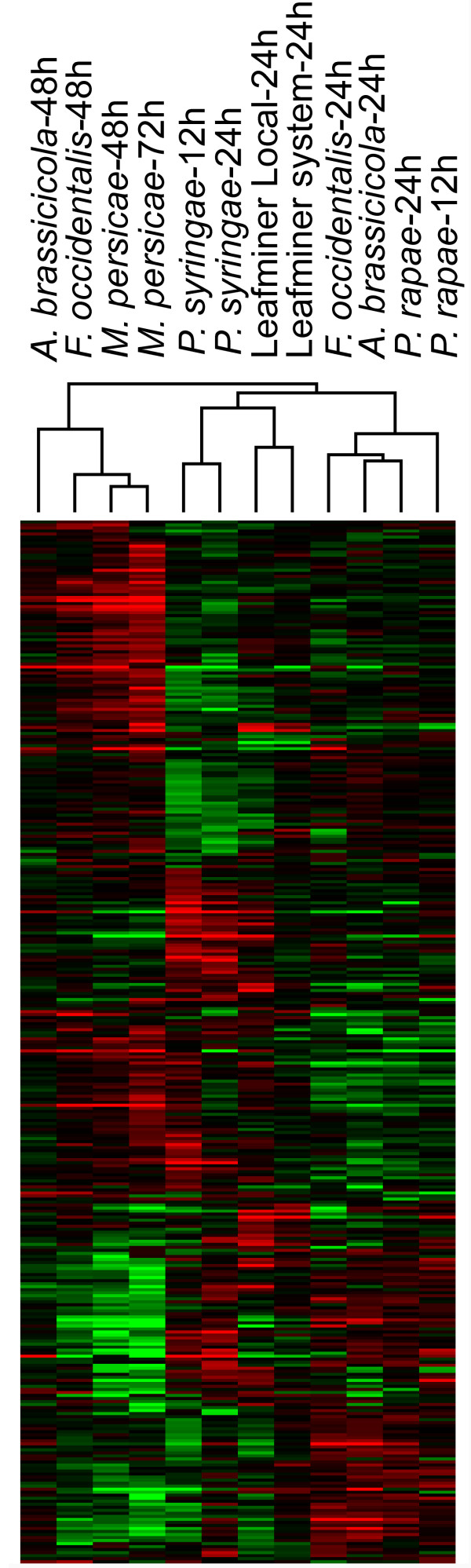
**Hierarchical clustering of *A. thaliana *differentially-expressed genes induced by leafminers or other organisms.** Organisms compared with leafminers were *Pseudomonas syringae* pv. *tomato*, *Alternaria brassicicola*, and the herbivorous insects *Pieris rapae, Myzus persicae,* and *Frankliniella occidentalis.*

To confirm the above findings, we analyzed published data from another study [20] in which researchers monitored expression patterns of *A. thaliana* in response to attack by a range of microbial pathogens and herbivorous insects with very different modes of action, including *Pseudomonas syringae* pv. *tomato*, *Alternaria brassicicola*, and the herbivorous insects *Pieris rapae, Myzus persicae,* and *Frankliniella occidentalis*. Using the same method described above, we performed hierarchical clustering on the differentially-expressed gene data from that study. The results clearly demonstrated that the expression pattern of leafminer-damaged *A. thaliana* was most closely correlated to the pattern induced by *Pseudomonas syringae* pv*. tomato*, a well-characterized microbial pathogen (Figure 5).

**Figure 6:**
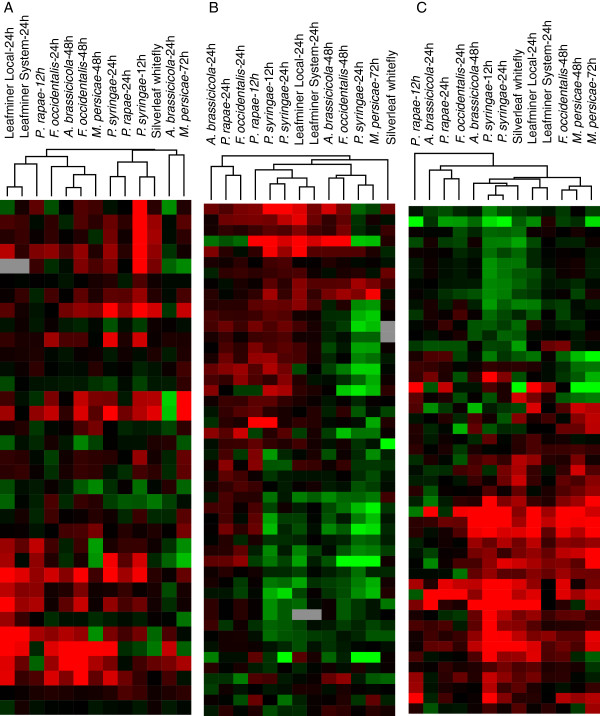
**Hierarchical clustering of three gene categories in *A. thaliana *induced by leafminers and other organisms.** Organisms compared with leafminers were *Pseudomonas syringae* pv. *tomato*, *Alternaria brassicicola*, and the herbivorous insects *Pieris rapae, Myzus persicae, Frankliniella occidentalis*, and *Bemisia tabaci* type B (SLWF). (**A**) Hierarchical clustering of some key marker genes of SA, JA, and ET pathways in *A. thaliana* induced by leafminers and other organisms. (**B**) Hierarchical clustering of genes influencing glucosinolate metabolism in *A. thaliana* induced by leafminers and other organisms. (**C**) Hierarchical clustering of some important genes related to plant response to biological attackers, including oxidative stress, cell wall biosynthesis and modification, photosynthesis, signal transduction, and nitrogen and carbohydrate metabolism in *A. thaliana* induced by leafminers and other organisms.

To further investigate correlations between plant defenses and different types of attackers, we compared expression in three important gene categories: 1) key marker genes of SA, JA, and ET pathways (Figure 6A, Additional file 7); 2) genes influencing metabolism of glucosinolate, an important secondary metabolite involved in plant interactions with pathogens and herbivores (Figure 6B, Additional file 8); and 3) some important genes related to plant response to biological attackers, including oxidative stress, cell wall biosynthesis and modification, photosynthesis, signal transduction, and nitrogen and carbohydrate metabolism (Figure 6C, Additional file 9). In *A. thaliana*, JA, SA, and ET signal pathway responses to leafminers were most similar to those of *Pieris rapae*: JA signal pathways were deeply up-regulated in response to both organisms, but SA and ET signals were only slightly affected (Additional file 7). On the other hand, *A. thaliana* glucosinolate metabolism (Figure 6B) and important plant physiology responses (Figure 6C) after leafminer attack closely resembled those following infestation by *Pseudomonas syringae* pv. *tomato*.

## Discussion

When we analyzed local and systemic expression patterns in leafminer-damaged *A. thaliana*, we found that there was a trade-off between the different signal pathways expressed. Many defense genes were up-regulated, whereas some stimulus response genes were down-regulated. After carefully analyzing these pathways, we found that the up-regulated genes were mainly involved in biotic stress response, while the down-regulated defense genes were related to abiotic stress. It can thus be seen that the plant defense system can deal with specific stimuli while simultaneously down-regulating other defense pathways to save energy.

Appropriate damage pattern recognition is very important for plants to express suitable defense genes. For example, the defense of *A. thaliana* to wounding is stronger than to *Pieris rapae* damage [10], because *P. rapae* can reduce tissue crushing and minimize cut leaf edges while removing maximum tissue mass. Indeed, two sets of variables play important roles in the host response: the exact nature of the physical injury and the extent to which elicitors are exposed to the host [21]. Many insects and pathogens try to escape plant defenses by minimizing these two parameters [10,11]. In this sense, the leafminer is not a good model of successful insect evolution, because the leaf-mining habit does not effectively diminish these two parameters [16,22]. Finally, it should be noted that disease incidence in leaf-mining insects is remarkably lower than in external-feeding insects [16]; this suggests that the similarity of gene expression patterns between leafminer-damaged and bacterial pathogen-damaged *A. thaliana* is not caused by leafminer-derived disease.

Plant transcriptome responses to different attackers may be influenced not only by the attacker species, but also by plant physiological conditions. For example, inbred *Solanum carolinense* seedlings show markedly weaker defenses to herbivores than do outbred seedlings [23]. When downloading data from TAIR databases and other published sources, we were therefore careful to select data from plants of the same genetic background and with similar physiological conditions and ages. Because responses of *A. thaliana* to leafminers and pathogens have similar patterns, just as their similar damage patterns we conclude that damage patterns of attackers play an important role in eliciting plant responses. Further investigation indicates secondary metabolite metabolism and some plant physiological responses, but not plant hormones, give rise to the defense pattern correlations among different attackers. In a previous study, *A. thaliana* response to silverleaf whitefly (*Bemisia tabaci* type B [SLWF]) involved regulation of several biotrophic pathogen defense pathways [7]; however, silverleaf whitefly damage strongly up-regulated SA signal pathways, whereas in our study leafminers primarily up-regulated JA signal pathways. Consequently, plant defense patterns to different attackers are shaped not only by interactions between different plant hormone signal pathways, but also different combinations of defense signal pathways, secondary metabolite metabolism, and important physiological responses.

## Conclusions

We studied transcriptome response characteristics of *A. thaliana* to leafminer (*L. huidobrensis*) damage, and then analyzed its relationships with the response to other attackers. We found that leafminers induced many defense- and metabolism-related genes, with some associated with pathogen defense. Further analysis indicated that the response pattern of *A. thaliana* to leafminers was most similar to the response pattern of *A. thaliana* to bacterial pathogens, which was consistent with the similar damage patterns of leafminers and bacterial pathogens. The type of damage pattern thus appears to be an important determinant of plant response patterns.

## Methods

### Plants and insects

Seeds of *A. thaliana* (Col-0 ecotype) derived from selfed progenies of an *Arabidopsis* plant were surface-sterilized for 15 min in 10% bleach, washed four times with sterile water, and plated on half-strength Murashige and Skoog [24] medium. Plants were stratified at 4°C for 2 d in darkness and then transferred to a phytotron set at 22°C under a 16-h light/8-h dark photoperiod (light intensity 120 μmol m^-2^ s^-1^). After two to three weeks, seedlings were transplanted into a mixed peat-vermiculite (1:2) potting medium and placed in a growth chamber at 22°C under a 16-h light/8-h dark cycle (light intensity 120 μmol m^-2^ s^-1^). Four-week-old *A. thaliana* seedlings were used in subsequent experiments.

Seeds of *Phaseolus vulgaris* L. (cv. Naibai; Haizhong Vegetable Market, Beijing, China) were individually sown in 12-cm-diameter plastic pots containing a mixed peat-vermiculite (3:1) potting medium in an environmental chamber. Bean plants with two fully-developed true leaves were used as leafminer reproductive hosts.

Pea leafminers (*L. huidobrensis*) used in experiments had been under continuous culture for three years in our laboratory. During the course of the experiments, newly-emerged leafminers were reared on 10% honey for 2 d to permit mating before release onto *A. thaliana* plants.

### Plant treatment and cDNA sample preparation

Approximately 50 mated *L. huidobrensis* adults were released for oviposition onto leaves of four-week-old *A. thaliana* plants in eight pots (four seedlings per pot). The *A. thaliana* seedlings were immature and had not yet begun to flower. Adult leafminers were removed within 4 h, by which time approximately half of the leaves had experienced damage. When leafminer larvae had reached the second instar stage, 96 h after oviposition, damaged leaves of *A. thaliana* containing leafminer larvae were collected for local damage analysis (LI); intact leaves adjacent to the damaged leaves on the same plant were collected for systemic damage analysis (SI). The samples were separately frozen in liquid nitrogen. Three replicate samples, each containing tissue from at least eight plants, were prepared for both LI and SI. Three control samples (H) were also prepared from leaves of same-aged healthy plants. Total RNAs were isolated from each replicate and control sample using an RNeasy Plant Mini Kit (Qiagen, Valencia, CA, USA). cDNA was synthesized from the isolated RNA following the manufacturer’s instructions (http://www.affymetrix.com/support/technical/manuals.affx). Expression Analysis Technical Manual For HT Array Plates Using the GeneChip® Array Station. 

### Microarray hybridization and data analysis

Affymetrix microarrays (*A. thaliana* ATH1 genome arrays) containing 22,810 probe sets were used in our experiments. Labeling and hybridization of the ATH1 microarrays (one sample per chip) was performed according to the manufacturer’s instructions (http://www.affymetrix.com/support/technical/manuals.affx). Expression Analysis Technical Manual For HT Array Plates Using the GeneChip® Array Station. Quality control was carried out according to Affymetrix microarray standards, which indicated that all samples met required criteria with respect to control signals, house-keeping gene signals, “present” percent numbers, and low background and noise values. The probe arrays were scanned and further analyzed using GENESPRING software (version 5.0; Silicon Genetics). Normalization per gene and per chip of the log2 values was performed to allow comparison of the three independent replicates performed for each set of experiments. In addition, normalization was performed separately for each experiment and plant tissue for all measurements using the flags “present”, “marginal”, or “absent” assigned by Affymetrix treatment of the arrays. However, only those transcripts that were declared “present” or “marginal” in at least two of three chips were taken into account. To identify differentially-expressed genes in each treatment, SAM analysis (Significance Analysis of Microarrays software package) was conducted on *A. thaliana* triplicate samples between treatments and controls using a q-value ≤ 0.05 and fold change ≥ 2 as cut-off criteria. We searched for GO information for the differently-expressed probe sets using EasyGO software (http://bioinformatics.cau.edu.cn/easygo/category_treeBrowse.html). For biological process searching, we applied χ*2* tests with a false discovery rate (FDR)-adjusted cutoff of *P* < 0.0001. Cluster 3.0/Treeview software [25] was used to group and display genes with similar expression profiles (http://rana.Stanford.EDU/software/). Hierarchical clustering using default options and the uncentered correlation similarity metric was performed on the normalized data. The microarray data in MIAME-compliant format have been deposited in the GEO database (GEO record number GSE38281).

### Downloading of microarray data from public databases

Transcriptome datasets used for expression cluster analysis of different damage patterns were downloaded from the Arabidopsis Information Resource (TAIR) public GeneChip database (ftp://ftp.arabidopsis.org/home/tair/Microarrays/Datasets/). Differential genes from each experiment were identified using the same method used for our own data. Detailed information regarding downloaded datasets from the TAIR microarray database is listed in Additional file 10. Other data was derived from the results of a published study [20].

### Quantitative real-time PCR

PCR reactions were performed in 20-μl reaction volumes that included 10 μl of 2X SYBR Premix EX Taq™ master mix (Takara, Kyoto, Japan), 0.25 μM each of gene-specific primers (Table 1), and 1 μl cDNA templates. The amplifications were carried out on an Mx 3000P detection system (Stratagene, La Jolla, CA, USA) with reaction conditions as follows: 10 s at 95°C, followed by 40 cycles of 5 s at 95°C, 20 s at 58°C, and 20 s at 72°C, and a final cycle of 30 s at 95°C, 30 s at 58°C, and 30 s at 95°C, yielding melting curves used to judge specificity of the PCR products. β-actin was used as a housekeeping gene. A standard curve was derived from serial dilutions to quantify the copy numbers of target mRNAs, and gene amounts were normalized to β-actin levels. Normalized values of each gene in the stressed samples were then divided by those from the untreated controls, and the folds were used as the relative levels of each gene. To correct for plate variation, the *lox* mRNA level of healthy *A. thaliana* grown at 22°C was quantified on each plate.

**Table 1:** Primers used for qRT-PCR to validate fold change of microarray hybridization

**Probe sets**	**Sense primer 5**^**′**^**-3**^**′**^	**Anti-sense primer 5**^**′**^**-3**^**′**^
259878_at	GAACACTTGCTTCTCAACTTATT	TCACCAACTCCACCTCCTA
260203_at	CACGGTCTTGCGGATACTTCTA	CGAGTCAACACCATAACCCTTT
266267_at	GAGAAGGGAGTAGAGGTTGC	TTTTGATCCATCTGTTTAGC
261037_at	GACAATACCCTTACGGTGGCT	GCTTCAACAATCTCCGCATCT
264886_at	CGCTATCAAATTCACAGG	AACAAAGAGCCATCCATAC
261470_at	TTACAACATCAACGCCCACT	TAACGACATCCATCACCACC
Actin	GGAAGGATCTGTACGGTAAC	TGTGAACGATTCCTGGACCT

## Competing interests

The authors declare that they have no competing interests.

## Authors’ contributions

SZ designed and carried out the experiments, analyzed the data, and wrote the paper; ZZ helped modify the manuscript; LK designed the experiments and modified the manuscript. All authors read and approved the final manuscript.

## Supplementary Material

Additional file 1 **qRT-PCR validation of gene expression from microarray hybridization.** The data are fold changes averaged over 3–4 repetitions, from both qRT-PCR and microarray hybridization. Click here for file

Additional file 2 **Gene Ontology (GO) term enrichment status for SI exclusively-regulated genes.** The graph displays term enrichment levels along with the GO term hierarchy within the “biological process” branch. The analysis was performed using EasyGO. Classification terms and their serial numbers are represented as rectangles. Numbers in brackets represent the total number of genes that may be involved in the corresponding biological processes. The color scale shows the *P*-value cutoff levels for each biological process. Deeper colors represent the more significant biological processes in the putative signal pathway. Click here for file

Additional file 3 **GO term enrichment of locally up-regulated genes in leafminer-damaged *A. thaliana*.** The graph displays term enrichment levels along with the GO term hierarchy within the “biological process” branch. The analysis was performed using EasyGO. Classification terms and their serial numbers are represented as rectangles. Numbers in brackets represent the total number of genes that may be involved in the corresponding biological processes. The color scale shows the *P*-value cutoff levels for each biological process. Deeper colors represent the more significant biological processes in the putative signal pathway. Click here for file

Additional file 4 **GO term enrichment of systemically up-regulated genes in leafminer-damaged *A. thaliana*.** The graph displays term enrichment levels along with the GO term hierarchy within the “biological process” branch. The analysis was performed using EasyGO. Classification terms and their serial numbers are represented as rectangles. Numbers in brackets represent the total number of genes that may be involved in the corresponding biological processes. The color scale shows the *P*-value cutoff levels for each biological process. Deeper colors represent the more significant biological processes in the putative signal pathway. Click here for file

Additional file 5 **GO term enrichment of locally down-regulated genes in leafminer-damaged *A. thaliana*.** The graph displays term enrichment levels along with the GO term hierarchy within the “biological process” branch. The analysis was performed using EasyGO. Classification terms and their serial numbers are represented as rectangles. Numbers in brackets represent the total number of genes that may be involved in the corresponding biological processes. The color scale shows the *P*-value cutoff levels for each biological process. Deeper colors represent the more significant biological processes in the putative signal pathway. Click here for file

Additional file 6 **GO term enrichment of systemically down-regulated genes in leafminer-damaged *A. thaliana*.** The graph displays term enrichment levels along with the GO term hierarchy within the “biological process” branch. The analysis was performed using EasyGO. Classification terms and their serial numbers are represented as rectangles. Numbers in brackets represent the total number of genes that may be involved in the corresponding biological processes. The color scale shows the *P*-value cutoff levels for each biological process. Deeper colors represent the more significant biological processes in the putative signal pathway. Click here for file

Additional file 7 **Fold changes of some key marker genes of SA, JA, and ET pathways in *A. thaliana *induced by leafminer and other organisms.** Click here for file

Additional file 8 **Fold changes of genes influencing glucosinolate metabolism in *A. thaliana *induced by leafminer or other organisms.** Click here for file

Additional file 9 **Fold changes of some important genes related to plant response to biological attackers, including oxidative stress, cell wall biosynthesis and modification, photosynthesis, signal transduction, and nitrogen and carbohydrate metabolism in *A. thaliana *induced by leafminer and other organisms.** Click here for file

Additional file 10 List of data downloaded from the TAIR microarray database. Click here for file

## References

[B1] Ellis C Turner JG The Arabidopsis mutant cev1 has constitutively active jasmonate and ethylene signal pathways and enhanced resistance to pathogens Plant Cell 2001 13 5 1025 1033 11340179 10.1105/tpc.13.5.1025 PMC135553

[B2] Delaney TP Uknes S Vernooij B Friedrich L Weymann K Negrotto D Gaffney T Gut-Rella M Kessmann H Ward E A central role of salicylic acid in plant disease resistance Science 1994 266 5188 1247 1250 10.1126/science.266.5188.1247 17810266

[B3] Turner JG Ellis C Devoto A The jasmonate signal pathway Plant Cell 2002 14 S153 S164 12045275 10.1105/tpc.000679 PMC151253

[B4] Doares SH Narvaezvasquez J Conconi A Ryan CA Salicylic-acid inhibits synthesis of proteinase-inhibitors in tomato leaves induced by systemin and jasmonic acid Plant Physiol 1995 108 4 1741 1746 12228577 10.1104/pp.108.4.1741 PMC157556

[B5] Reymond P Farmer EE Jasmonate and salicylate as global signals for defense gene expression Curr Opin Plant Biol 1998 1 5 404 411 10.1016/S1369-5266(98)80264-1 10066616

[B6] Zheng S-J Dicke M Ecological genomics of plant-insect interactions: from gene to community Plant Physiol 2008 146 3 812 817 10.1104/pp.107.111542 18316634 PMC2259077

[B7] Kempema LA Cui X Holzer FM Walling LL Arabidopsis transcriptome changes in response to phloem-feeding silverleaf whitefly nymphs. Similarities and distinctions in responses to aphids Plant Physiol 2007 143 2 849 865 17189325 10.1104/pp.106.090662 PMC1803730

[B8] Cheong YH Chang H-S Gupta R Wang X Zhu T Luan S Transcriptional profiling reveals novel interactions between wounding, pathogen, abiotic stress, and hormonal responses in Arabidopsis Plant Physiol 2002 129 2 661 677 10.1104/pp.002857 12068110 PMC161692

[B9] Korth KL Dixon RA Evidence for chewing insect-specific molecular events distinct from a general wound response in leaves Plant Physiol 1997 115 4 1299 1305 12223872 10.1104/pp.115.4.1299 PMC158595

[B10] Reymond P Weber H Damond M Farmer EE Differential gene expression in response to mechanical wounding and insect feeding in Arabidopsis Plant Cell 2000 12 5 707 720 10810145 10.1105/tpc.12.5.707 PMC139922

[B11] Moran PJ Thompson GA Molecular responses to aphid feeding in Arabidopsis in relation to plant defense pathways Plant Physiol 2001 125 2 1074 1085 10.1104/pp.125.2.1074 11161062 PMC64906

[B12] Thompson GA Goggin FL Transcriptomics and functional genomics of plant defence induction by phloem-feeding insects J Exp Bot 2006 57 4 755 766 10.1093/jxb/erj135 16495409

[B13] Wei JN Zhu JW Kang L Volatiles released from bean plants in response to agromyzid flies Planta 2006 224 2 279 287 10.1007/s00425-005-0212-x 16404576

[B14] Parrella MP Bethke JA Biological Studies of Liriomyza-Huidobrensis (Diptera, Agromyzidae) on Chrysanthemum, Aster, and Pea J Econ Entomol 1984 77 2 342 345

[B15] Parrella MP Biology of Liriomyza Annu Rev Entomol 1987 32 201 224 10.1146/annurev.en.32.010187.001221

[B16] Connor EF Taverner MP The evolution and adaptive significance of the leaf-mining habit Oikos 1997 79 1 6 25 10.2307/3546085

[B17] Wei JN Zou L Kuang RP He LP Influence of leaf tissue structure on host feeding selection by pea leafminer Liriomyza huidobrensis (Diptera: Agromyzidae) Zool Stud 2000 39 4 295 300

[B18] Ye J Fang L Zheng H Zhang Y Chen J Zhang Z Wang J Li S Li R Bolund L WEGO: a web tool for plotting GO annotations Nucleic Acids Res 2006 34 Web Server issue W293 W297 16845012 10.1093/nar/gkl031 PMC1538768

[B19] Zhou X Su Z EasyGO: gene ontology-based annotation and functional enrichment analysis tool for agronomical species BMC Genomics 2007 8 246 10.1186/1471-2164-8-246 17645808 PMC1940007

[B20] De Vos M Van Oosten VR Van Poecke RM Van Pelt JA Pozo MJ Mueller MJ Buchala AJ Metraux JP Van Loon LC Dicke M Signal signature and transcriptome changes of Arabidopsis during pathogen and insect attack Mol Plant Microbe Interact 2005 18 9 923 937 10.1094/MPMI-18-0923 16167763

[B21] Farmer EE Adding injury to insult: pathogen detection and responses Genome Biol 2000 1 2 1012.1 1012.3 10.1186/gb-2000-1-2-reviews1012 PMC138849 11178234

[B22] Engelbrecht L Orban U Heese W Leaf-miner caterpillars and cytokinins in the green Islands of Autumn leaves Nature 1969 223 5203 319 321 10.1038/223319a0

[B23] Kariyat RR Mena-Alí J Forry B Mescher MC Moraes CMD Stephenson AG Inbreeding, herbivory, and the transcriptome of solanum carolinense Entomol Exp Appl 2012 144 134 144 10.1111/j.1570-7458.2012.01269.x

[B24] Murashige T Skoog F A Revised Medium for rapid growth and bio asssays with tobacco tissue cultures Physiol Plant 1962 15 3 473 497 10.1111/j.1399-3054.1962.tb08052.x

[B25] Eisen MB Spellman PT Brown PO Botstein D Cluster analysis and display of genome-wide expression patterns Proc Natl Acad Sci USA 1998 95 25 14863 14868 10.1073/pnas.95.25.14863 9843981 PMC24541

