# Supplementary figures and images for: Transcriptome response analysis of Arabidopsis thaliana to leafminer (Liriomyza huidobrensis)
Source: BMC Plant Biol. 2012 Dec 11;12:234. doi: 10.1186/1471-2229-12-234 (PMC3564828; doi:10.1186/1471-2229-12-234)

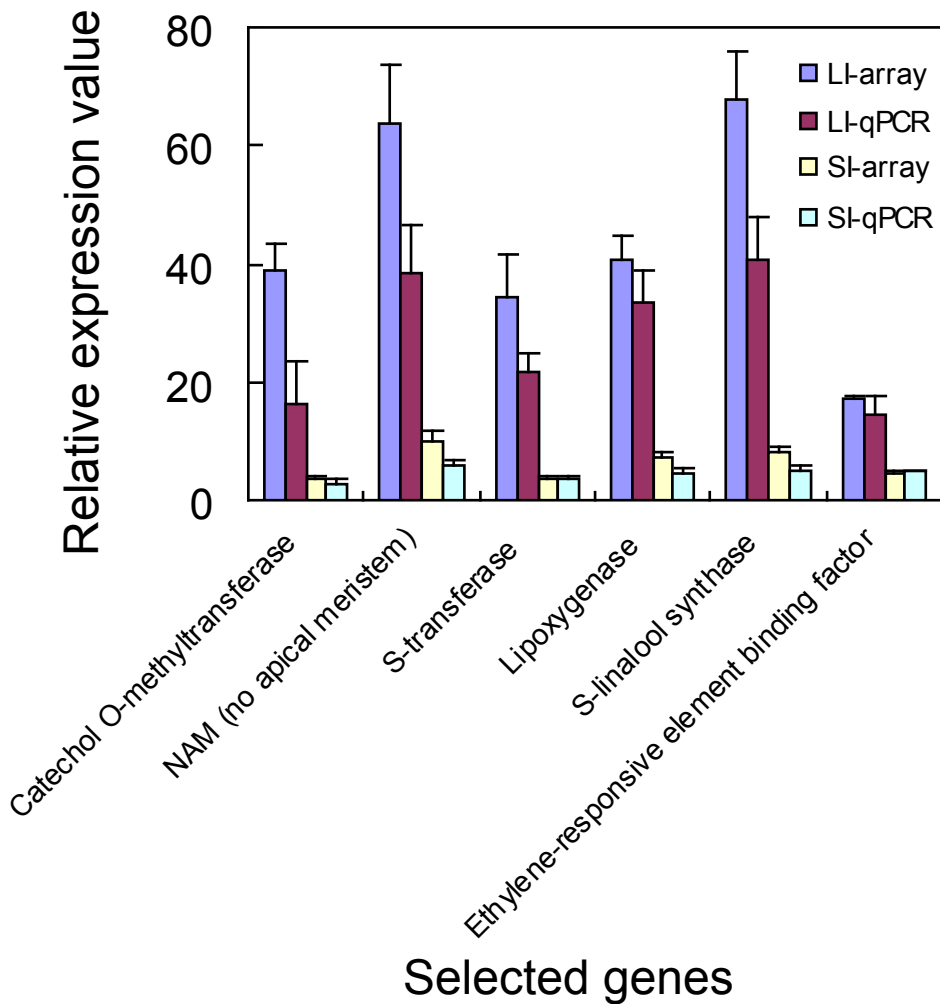

Supplement: Additional file 1 — qRT-PCR validation of gene expression from microarray hybridization. The data are fold changes averaged over 3–4 repetitions, from both qRT-PCR and microarray hybridization. [file 1471-2229-12-234-S1.pdf]

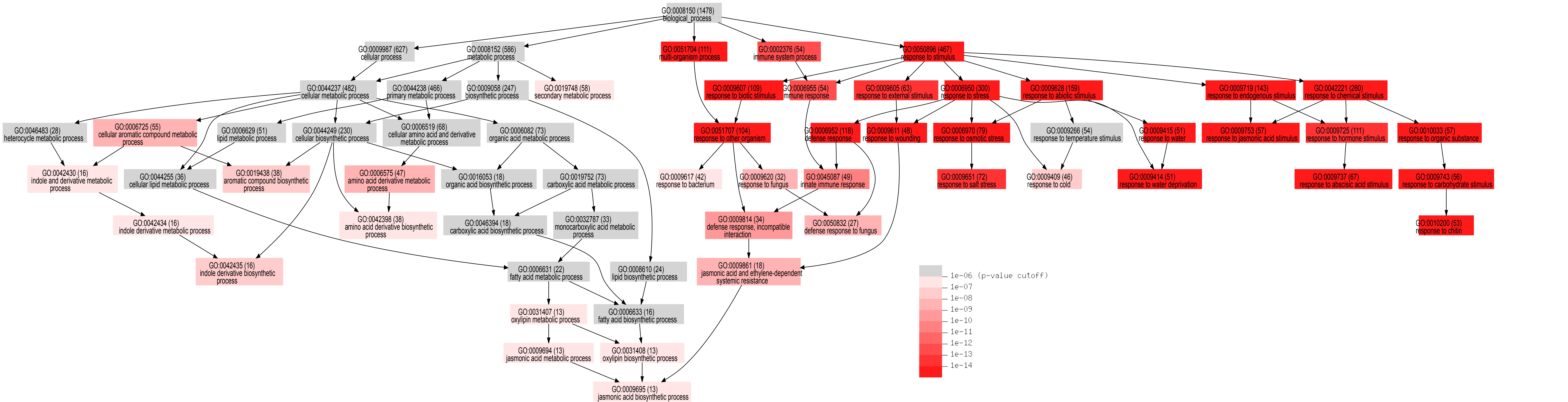

Supplement: Additional file 3 — GO term enrichment of locally up-regulated genes in leafminer-damaged A. thaliana. The graph displays term enrichment levels along with the GO term hierarchy within the “biological process” branch. The analysis was performed using EasyGO. Classification terms and their serial numbers are represented as rectangles. Numbers in brackets represent the total number of genes that may be involved in the corresponding biological processes. The color scale shows the P-value cutoff levels for each biological process. Deeper colors represent the more significant biological processes in the putative signal pathway. [file 1471-2229-12-234-S3.pdf]

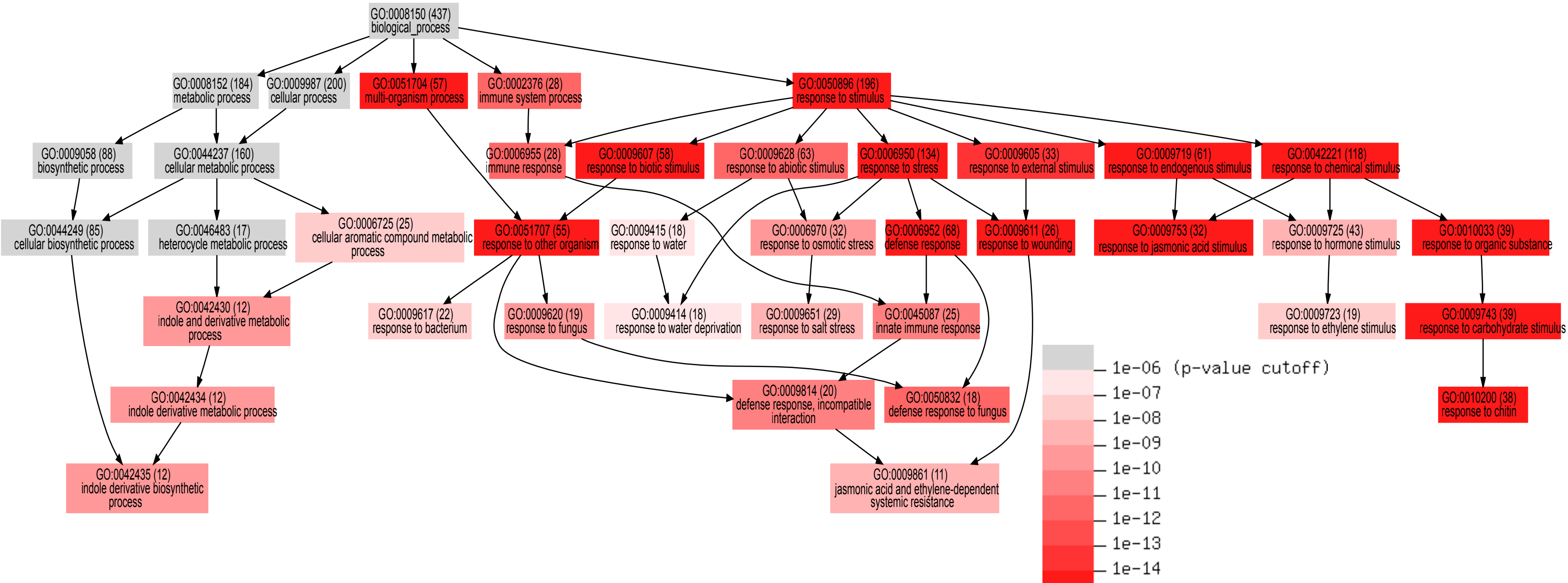

Supplement: Additional file 4 — GO term enrichment of systemically up-regulated genes in leafminer-damaged A. thaliana. The graph displays term enrichment levels along with the GO term hierarchy within the “biological process” branch. The analysis was performed using EasyGO. Classification terms and their serial numbers are represented as rectangles. Numbers in brackets represent the total number of genes that may be involved in the corresponding biological processes. The color scale shows the P-value cutoff levels for each biological process. Deeper colors represent the more significant biological processes in the putative signal pathway. [file 1471-2229-12-234-S4.pdf]

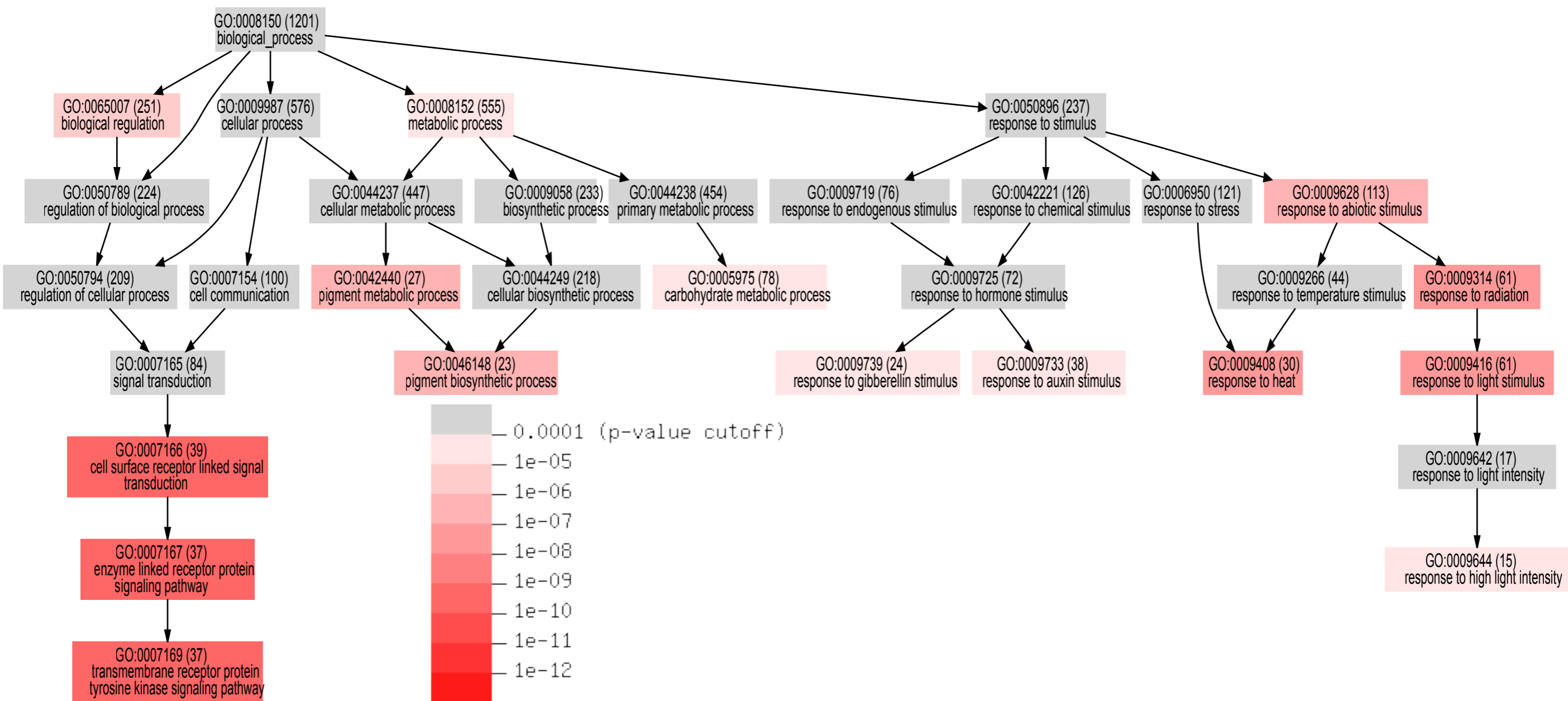

Supplement: Additional file 5 — GO term enrichment of locally down-regulated genes in leafminer-damaged A. thaliana. The graph displays term enrichment levels along with the GO term hierarchy within the “biological process” branch. The analysis was performed using EasyGO. Classification terms and their serial numbers are represented as rectangles. Numbers in brackets represent the total number of genes that may be involved in the corresponding biological processes. The color scale shows the P-value cutoff levels for each biological process. Deeper colors represent the more significant biological processes in the putative signal pathway. [file 1471-2229-12-234-S5.pdf]
