# Supplementary material for: Transcriptome response analysis of Arabidopsis thaliana to leafminer (Liriomyza huidobrensis)
Source: BMC Plant Biol. 2012 Dec 11;12:234. doi: 10.1186/1471-2229-12-234 (PMC3564828; doi:10.1186/1471-2229-12-234)
Supplement: Additional file 2 — Gene Ontology (GO) term enrichment status for SI exclusively-regulated genes. The graph displays term enrichment levels along with the GO term hierarchy within the “biological process” branch. The analysis was performed using EasyGO. Classification terms and their serial numbers are represented as rectangles. Numbers in brackets represent the total number of genes that may be involved in the corresponding biological processes. The color scale shows the P-value cutoff levels for each biological process. Deeper colors represent the more significant biological processes in the putative signal pathway. [file 1471-2229-12-234-S2.pdf]

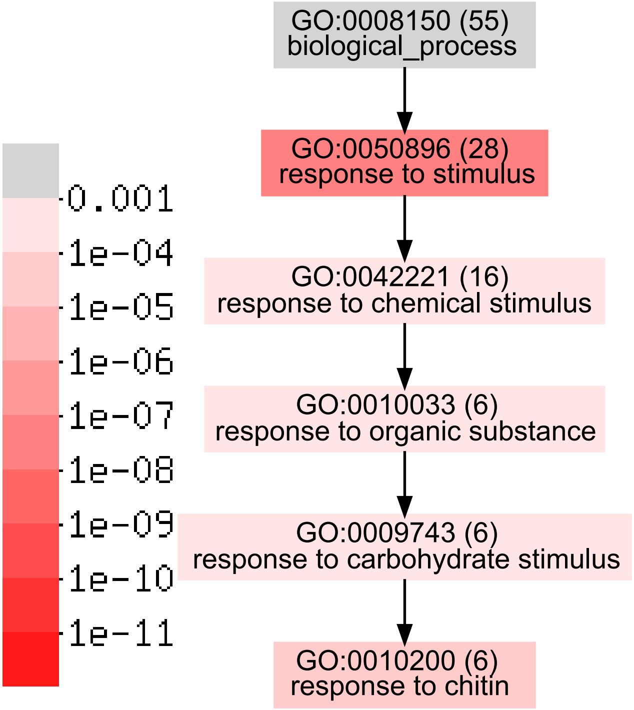
0.001
1e-04
1e-05
1e-06
1e-07
1e-08
1e-09
1e-10
1e-11
GO:0008150 (55)
biological_process
GO:0050896 (28)
response to stimulus
GO:0042221 (16)
response to chemical stimulus
GO:0010033 (6)
response to organic substance
GO:0009743 (6)
response to carbohydrate stimulus
GO:0010200 (6)
response to chitin
