# Supplementary material for: Transcriptome response analysis of Arabidopsis thaliana to leafminer (Liriomyza huidobrensis)
Source: BMC Plant Biol. 2012 Dec 11;12:234. doi: 10.1186/1471-2229-12-234 (PMC3564828; doi:10.1186/1471-2229-12-234)
Supplement: Additional file 6 — GO term enrichment of systemically down-regulated genes in leafminer-damaged A. thaliana. The graph displays term enrichment levels along with the GO term hierarchy within the “biological process” branch. The analysis was performed using EasyGO. Classification terms and their serial numbers are represented as rectangles. Numbers in brackets represent the total number of genes that may be involved in the corresponding biological processes. The color scale shows the P-value cutoff levels for each biological process. Deeper colors represent the more significant biological processes in the putative signal pathway. [file 1471-2229-12-234-S6.pdf]

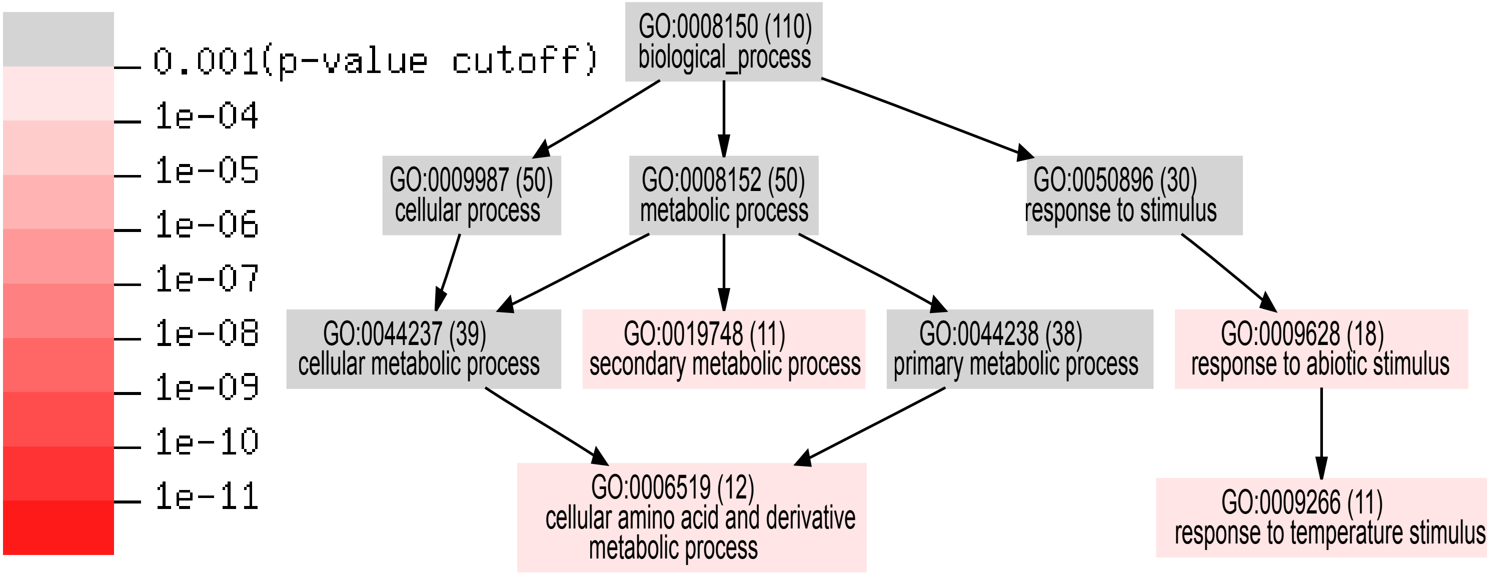
0.001(p-value cutoff)
1e-04
1e-05
1e-06
1e-07
1e-08
1e-09
1e-10
1e-11
GO:0008150 (110)
biological_process
GO:0009987 (50)
cellular process
GO:0008152 (50)
metabolic process
GO:0050896 (30)
response to stimulus
GO:0044237 (39)
cellular metabolic process
GO:0019748 (11)
secondary metabolic process
GO:0044238 (38)
primary metabolic process
GO:0009628 (18)
response to abiotic stimulus
GO:0006519 (12)
cellular amino acid and derivative metabolic process
GO:0009266 (11)
response to temperature stimulus
